# Microencapsulated Isoniazid-Loaded Metal–Organic Frameworks for Pulmonary Administration of Antituberculosis Drugs

**DOI:** 10.3390/molecules26216408

**Published:** 2021-10-23

**Authors:** Cristina Fernández-Paz, Estefanía Fernández-Paz, Pablo Salcedo-Abraira, Sara Rojas, Sheila Barrios-Esteban, Noemi Csaba, Patricia Horcajada, Carmen Remuñán-López

**Affiliations:** 1 Nanobiofar Group, Department of Pharmacology, Pharmacy & Pharmaceutical Technology, Faculty of Pharmacy, University of Santiago de Compostela, Campus Vida, 15782 Santiago de Compostela, Galicia, Spain; cristina.fernandez.paz@rai.usc.es (C.F.-P.); estefania.fernandez.paz@rai.usc.es (E.F.-P.); 2 Advanced Porous Materials Unit (APMU), IMDEA Energy Institute, Av. Ramón de la Sagra, 3, 28035 Móstoles, Madrid, Spain; pablo.salcedo@imdea.org (P.S.-A.); srojas@ugr.es (S.R.); patricia.horcajada@imdea.org (P.H.); 3 Nanobiofar Group-Natural Polymers and Biomimetics (NPNB) Group, Center of Research in Molecular Medicine and Chronic Diseases (CiMUS), University of Santiago de Compostela, Campus Vida, 15706 Santiago de Compostela, Galicia, Spain; sheila.barrios@rai.usc.es (S.B.-E.); noemi.csaba@usc.es (N.C.)

**Keywords:** A549 cells, isoniazid, mannitol, metal–organic frameworks, microencapsulation, pulmonary administration, tuberculosis

## Abstract

Tuberculosis (TB) is an infectious disease that causes a great number of deaths in the world (1.5 million people per year). This disease is currently treated by administering high doses of various oral anti-TB drugs for prolonged periods (up to 2 years). While this regimen is normally effective when taken as prescribed, many people with TB experience difficulties in complying with their medication schedule. Furthermore, the oral administration of standard anti-TB drugs causes severe side effects and widespread resistances. Recently, we proposed an original platform for pulmonary TB treatment consisting of mannitol microspheres (Ma MS) containing iron (III) trimesate metal–organic framework (MOF) MIL-100 nanoparticles (NPs). In the present work, we loaded this system with the first-line anti-TB drug isoniazid (INH) and evaluated both the viability and safety of the drug vehicle components, as well as the cell internalization of the formulation in alveolar A549 cells. Results show that INH-loaded MOF (INH@MIL-100) NPs were efficiently microencapsulated in Ma MS, which displayed suitable aerodynamic characteristics for pulmonary administration and non-toxicity. MIL-100 and INH@MIL-100 NPs were efficiently internalized by A549 cells, mainly localized in the cytoplasm. In conclusion, the proposed micro-nanosystem is a good candidate for the pulmonary administration of anti-TB drugs.

## 1. Introduction

Currently, TB continues to be one of the principal 10 causes of death worldwide [1,2], and is the second leading cause of death (1.5 million people in the world per year [3]) due to an infectious disease, behind SARS-CoV-2 (>1.9 million deaths in 2020 [4]). TB is mainly caused by the bacteria *Mycobacterium tuberculosis* [5] and principally affects the lungs but also can be spread to other organs (e.g., productive cough, fatigue, fever, malaise, night sweats, and weight loss) [6].

Once in the body, alveolar macrophages are a growth niche in which the bacilli reside and multiply. The conventional TB therapy recommended by the World Health Organization (WHO) consists of a difficult daily oral multidrug regimen that is prolonged in time (often lasts up to 2 years), in which the treatment during the first two months is with INH, rifampicin, ethambutol, and pyrazinamide (first-line anti-TB drugs), and the following four months with INH and rifampicin [7]. Although the treatment efficacy was estimated to be of 85% [8], there are several factors that can negatively affect the success rate, such as: (i) the inappropriate use of anti-TB drugs (e.g., employed medicines of deficient quality or bad storage); (ii) low stability and poor oral absorption of anti-TB drugs; (iii) important first-pass metabolism of anti-TB drugs, making difficult to reach the granulomas and penetrate into the bacteria; and (iv) a too-long treatment duration (often lasts up to 2 years) that is generally associated with important undesirable side effects (e.g., depression, psychosis, kidney impairment, or resistances [8]). Therefore, a controlled and targeted release of anti-TB drugs should be able to overcome these drawbacks and achieve a more effective treatment of TB.

The pulmonary route is suitable to administrate either small or large molecules for local or systemic treatments [9,10,11] by using aerosols [12]. In this regard, it has attracted great interest due to its advantages for the local delivery of anti-TB drugs [12], such as: (i) its non-invasive and pain-free way of administration that favors the comfort and dosing compliance of the patient [13]; (ii) direct administration at the site of action, reducing the administered dose and potential side effects; (iii) low enzymatic activity in comparison with other mucosal surfaces, avoiding, for example, the gastrointestinal degradation inherent to oral administration; and (iv) absence of the hepatic first-pass effect [14]. The pulmonary route is especially attractive for TB treatment in comparison with the traditional oral and parenteral routes, as it increases the local drug concentration, thus improving the pharmacological action and decreasing the drug systemic dosage, which are associated to side effects and to the risk of resistant-strain development [15,16,17].

Nano and microsystems arouse great interest for the delivery of active ingredients (AIs) directly to the lung to treat pulmonary diseases such as cystic fibrosis or TB, and also for gene therapy, as they can help to overcome the difficulties associated with their pulmonary administration. Additionally, the direct administration of AIs to the lung makes it possible to optimize their therapeutic effect in comparison with traditional treatments administered orally. Different types of nanosystems (NPs, solid lipid NPs (SLN), nanocapsules (NCs), nanoemulsions, liposomes, and micelles) and microsystems (microparticles and MS) [18,19,20,21,22,23] were investigated; among them, the MOFs stood out. They are crystalline hybrid materials of high porosity, which are formed by the association of inorganic subunits (cations, clusters, chains, etc.) and polydentate organic ligands (carboxylates, azolates, etc.) [24]. It is worthy to note that the presence of metals in the structure of some MOFs have antibacterial activity and a synergistic effect with antibacterial drugs; therefore, these MOFs have great potential for anti-TB therapy. This is the case of MOFs containing silver and gold [25]. MOFs (unlike other rigid NPs) are tunable, can be functionalized, have an adjustable porosity and high drug loads, and are thermo-resistant. Particularly, nanoscaled MOFs (nanoMOFs) have proven to be excellent platforms for the delivery of drugs through different routes such as the intravenous or cutaneous route [26]. The active compounds can be adsorbed or entrapped inside the pores or can be a constituent of the MOF itself as a biologically active building block. MOFs can transport large quantities of different AIs (drugs, nucleic acids, enzymes, etc.) and release them in a controlled manner [24]. Mainly mesoporous iron (III) trimesate, named MIL-100(Fe) (MIL = Material of Institute Lavoisier) [19], is a promising candidate as a drug delivery vehicle since: (i) it can be synthetized at difference sizes (from 80 nm to some microns) [24]; (ii) is highly porous (Brunauer, Emmett, and Teller (BET) surface area ~2000 m^2^·g^−1^, Vp = 1.2 cm^3^·g^−1^; with mesoporous voids of 2.5 and 2.9 nm accessible through microporous windows ca. 4.8–5.8 and 8.6 Å, respectively), which allows for the encapsulation of important drug doses; and (iii) is biodegradable and non-toxic, as it has not shown signs of toxicity after pulmonary [19] or intravenous [27] administration to rats. To prepare an optimal pulmonary formulation it is important to know where the drug must be deposited. In this sense, to achieve the deep lung, the aerodynamic diameter should be between 1 and 5 µm [14,28]. However, good distributions into the deep lung are achieved with particles lower in size and presenting hollow structure [29,30]. Regarding to the nature of inhaled formulations, dry powders are generally preferred over their liquid counterparts since: (i) they present a higher stability [31] and drug bioavailability [32]; (ii) are easily stored; and (iii) are easy to handle since, for example, the nebulization systems require special equipment [13,33]. Taking into account the fact that the MIL-100(Fe) NPs present a size of around 150 nm, it is not enough to reach the deep lung. In this regard, one efficient strategy can be the microencapsulation of MIL-100 NPs in MS with suitable aerodynamic properties to achieve the deep lung. The most common excipients used for the preparation of MS are the saccharides, as they are biocompatible, biodegradable, easily functionalizable, and cheap [34].

In this work, we microencapsulated INH@MIL-100 NPs by spray-drying in order to improve their stability [35] and their aerosolization pattern [36,37,38] to achieve a deposition and delivery in the lower respiratory tract [39,40]. Different biocompatible polysaccharides were initially spray-dried (obtaining control MS) to select the best excipient for the microencapsulation. The encapsulation of the INH first-line anti-TB drug was carried out in the nanoscaled MIL-100 MOFs, fully characterizing the resulting drug-loaded nanosystem (INH@MIL-100). Then, INH@MIL-100 NPs were microencapsulated in the MS of the selected excipient. The resulting micro-nanoMOFs were investigated to guarantee their biocompatibility with the pulmonary epithelium and the cell internalization of NPs in adenocarcinoma human alveolar basal epithelial cells (A549 cells) was examined. The developed Ma-INH@MIL-100 MS exhibited suitable aerodynamic properties for pulmonary administration. In addition, MIL-100 and INH@MIL-100 NPs, as well as the Ma excipient, were non-toxic for A549 cells, as the NPs were efficiently captured by these cells and the nanocarriers were found mainly in the cytoplasm. 

It is important to bear in mind that in certain cases where TB is advanced, pulmonary fibrosis can occur. If the lung tissue is fibrotic [41], the normal lung function is impaired. This modified functionality causes the diminishing of the iron uptake of the macrophages [42] and causes a low amount of phagocytosed iron to be less metabolized. Therefore, greater amounts of iron accumulate in the lung. This excess iron levels increases the activity of prolyl hydroxylase, an important enzyme involved in the synthesis [43] and maturation [44] of collagen, and, in consequence, worsens the fibrotic disease. The elevated iron levels can also increase the proliferation of pro-inflammatory cytokines. Hence, we did not rule out that in these circumstances, the pulmonary fibrosis may develop further after administration of MOFs containing iron. Therefore, Ma-INH@MIL-100 MS are more interesting for the early treatment of TB. We have recently reported the preparation of Ma MS containing MIL-100(Fe) MOFs, which have adequate aerodynamic properties to reach the deep lung after intratracheal administration to rats and are susceptible to being phagocytosed by alveolar macrophages. Therefore, Ma-INH@MIL-100 MS emerge as promising candidates to combat certain infectious diseases, such as TB, by the pulmonary route.

## 2. Results and Discussion

### 2.1. INH Encapsulation

**Figure 1:**
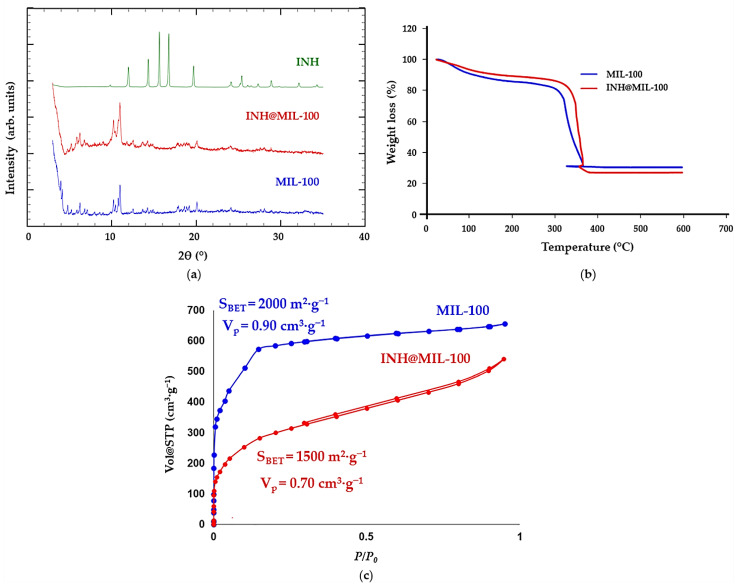
(**a**) PXRD patterns; (**b**) TGA; and (**c**) N_2_ sorption isotherms at 77 K of MIL-100 and INH@MIL-100 nanomaterials. PXRD pattern of the free INH has been included for comparison in Figure 1a. Close and open symbols in Figure 1c indicate adsorption and desorption branches, respectively.

The previously reported outstanding results in the efficient in vivo release of MIL-100 NPs uniformly along the lungs, reaching the bronchioles and alveoli [19], prompted us to explore the incorporation and posterior release of the first-line anti-TB drug INH (7.3 × 4.6 × 3.0 Å^3^) in detail. INH was successfully encapsulated into the porosity of the MIL-100 NPs with a two-step impregnation method of the porous material in an aqueous solution of INH, reaching a drug loading of 29.5 ± 2.21 wt%. Powder X-ray diffraction (PXRD) patterns evidenced that the drug-encapsulation process did not alter the crystalline structure of the porous material (Figure 1a). The absence of Bragg peaks corresponding to free INH rules out the presence of free recrystallized drugs outside the pores of MIL-100. The drug content was estimated by combining high-performance liquid chromatography (HPLC) and thermogravimetric analysis (TGA; Figure 1b). This INH loading is excellent in comparison with that obtained for MIL-101-NH_2_(Fe) (12 wt%) in other anterior studies [45] and remains in the range of previously incorporated drugs in MIL-100 MOF (e.g., 25 wt% of acetylsalicylic acid [46] and 31 wt% of ibuprofen [47]).

Furthermore, the incorporation of the anti-TB drug within the cavities of MIL-100 NPs was demonstrated by the dramatic reduction of the N_2_ sorption capacity of both the MOF and pore size (Figure 1c). In the INH@MIL-100 NPs, there was still some residual porosity after the drug encapsulation process (SBET = 1500 m^2^·g^−1^, pore volume-Vp = 0.70 cm^3^·g^−1^). To shed some light on the influence of porosity on drug adsorption, we estimated the volume occupied by each INH molecule inside the MOF by considering the variation of the MOF pore volume after the drug encapsulation and the total amount of loaded drug using a previously reported procedure described by Rojas et al. [47]. The higher INH occupancy volume in MIL-100 compared to the INH-free MIL-100 volume (435 vs. 105 Å^3^, as estimated under vacuum by ChemDraw) is related to the partial pores’ occupancy, as confirmed by the important remaining porosity after INH insertion. This suggests that the maximum drug loading is not reached in MIL-100 or that some cages are not accessible to the drug but only to N_2_. In fact, the adsorption of the INH might preferentially occur in the larger cages of MIL-100 (accessible via hexagonal windows of ~8.6 Å). In contrast, the dimensions of INH might prevent its crossing through the pentagonal windows (ca. 4.8–5.8 Å; note that van der Waals radii have been considered), in agreement with the selective location of other drugs in MIL-100 (e.g., ibuprofen) [48]. Finally, if one excludes the drug location within the smaller pores, a more realistic volume of the MIL-100 occupied by INH (44%) is estimated, corresponding to an INH occupancy volume of 191.4 Å^3^ (much closer to the free molecule 105 Å^3^).

**Figure 2:**
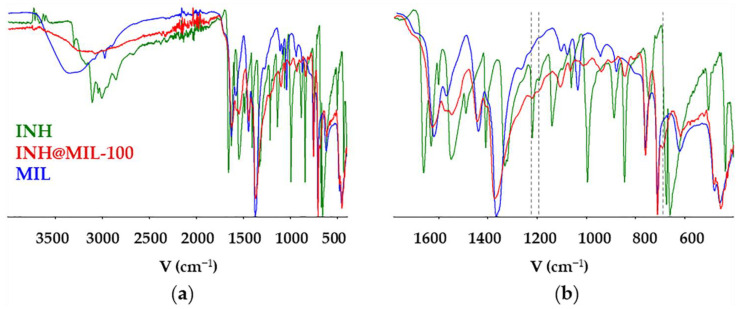
FTIR spectra of INH, INH@MIL-100, and MIL-100 materials with a (**a**) wider spectrum range, approximately from 4000 to 400 υ(cm^−1^). (**b**) A part of these spectra are around 1800 to 400 υ(cm^−1^).

Fourier transform infrared (FTIR) spectra of INH@MIL-100 confirmed the presence of INH in the MOF NPs through the presence of some bands corresponding to the drug (Figure 2). It can be seen, for instance, the ν(ring C-C-H) asymmetric and symmetric bending 1217, 1195 cm^−1^, and 843 cm^−1^, respectively; and ν(C-C=O) 689 cm^−1^ [49]. Furthermore, the FTIR spectrum of INH@MIL-100 NPs showed a shift in the wavelengths in comparison with the empty MIL-100 characteristic bands: ν(C-O) band of carboxylate groups (from 1630 to 1624 cm^−1^) and both the asymmetric and symmetric vibrational band characteristics of the -O-C-O- group (from 1449 to 1443 cm^−1^ and from 1378 to 1371 cm^−1^) [50]. These results suggest the formation of interactions between the drug and the hybrid framework.

### 2.2. Preparation and Characterization of MS

**Figure 3:**
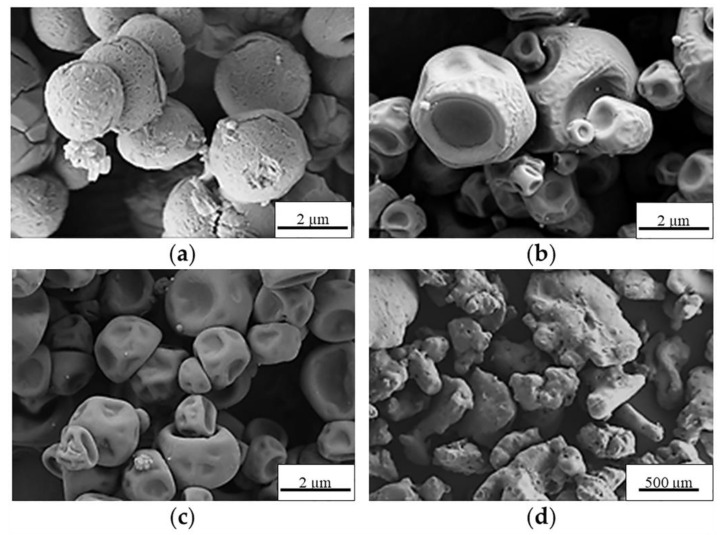
SEM microphotographs of the control MS of (**a**) Ma, (**b**) α-CD, (**c**) Dex, and (**d**) Tre.

A preliminary spray-drying screening study of the preparation of control MS (without NPs) was initially performed, employing the aqueous solutions of seven excipients: D-mannitol (Ma), α-cyclodextrin (α-CD), dextran (Dex), D-(+)-mannose (Man), D-(+)-trehalose dihydrate (Tre), D-sorbitol (Sor), and D-(−)-fructose (Fru). The resulting powders were characterized, with special attention provided to the process yield (PY), morphology, and apparent density (Table 1). In this regard, high PY was obtained for α-CD and Ma (74.0 ± 3.0 and 58.5 ± 3.5 wt%, respectively), in contrast to the low PY found for Tre and Dex (27.0 ± 1.0 and 10.5 ± 1.5 wt%, respectively). Sor, Man, and Fru did not allow for the recovery of measurable powder amounts; therefore, they were discarded. Conversely, SEM images (Figure 3) showed that the MS morphology was highly dependent on the used excipient. Ma MS were well-defined non-aggregated spherical particles with a continuous smooth surface (Figure 3a), whereas α-CD MS and Dex MS were more irregular and with a heterogeneous surface (Figure 3b and Figure 3c, respectively). In contrast, the resulting Tre structures did not present qualities for pulmonary administration because they resulted in agglomerates. One could expect that regular spherical Ma MS, with fewer contact points between particles and less friction between them [51], will present improved flow properties, facilitating their pulmonary administration. 

The higher apparent density obtained for Ma MS (0.43 ± 0.01 g·cm^−3^) compared to the α-CD MS (0.36 ± 0.01 g·cm^−3^; Table 1) can be explained by their different surface morphologies. In fact, the apparent density of α-CD MS was very similar to that of locust bean gum MS (around 0.37 g·cm^−3^), which present analogous surface morphology [52]. The apparent densities of Dex MS and Tre MS could not be determined due to their low PY (9–28%). Ma was the only excipient that resulted in control MS with good handling properties, probably because Ma allows for obtaining powders with low humidity content [53], which would also facilitate the in vivo aerosolization. Considering the advantages of Ma MS dry powder, such as elevated spray-drying PY as well as regular spherical particles with a priori improved flow properties and better handling (drier powder), Ma was selected as the excipient for INH@MIL-100 NPs microencapsulation. Ma has been extensively used in aerosolization [54]. It is accepted by regulatory authorities for its use in inhalation pharmaceutical products; it has suitable characteristics for spray-drying, allowing for the easy adaptation of the morphology and size of the particles [55]; and it improves the handling of the powder formulation as compared to other excipients, such as lactose [56]. In addition, Ma dry-powder facilitates the mucocilliary clearance and the cough in people with asthma and mucocilliary dysfunction [57] due to its osmotic effect, which improves the hydration of the mucus.

**Figure 4:**
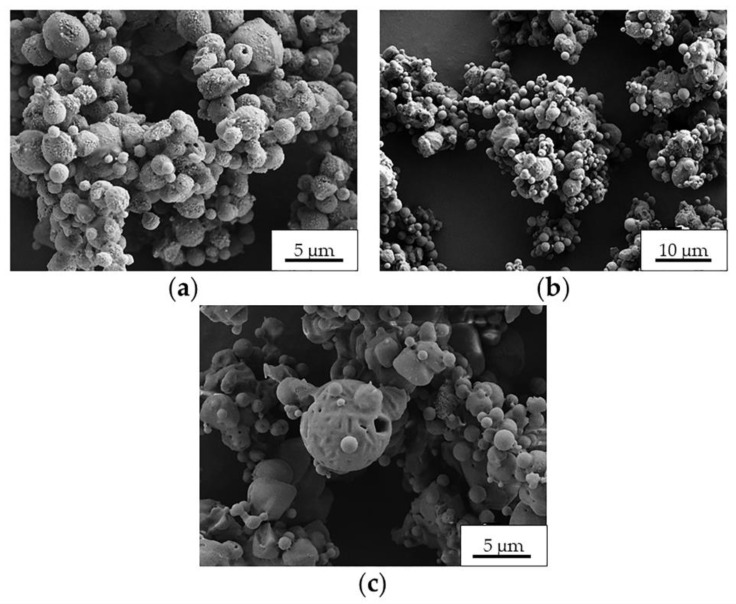
SEM microphotographs of Ma-MIL-100 MS prepared with (**a**) 1:5, (**b**) 1:10, and (**c**) 1:100 MIL-100:Ma ratios.

Once Ma was selected as the best excipient, the preparation of Ma-INH@MIL-100 MS was performed in consideration of various parameters, such as the shear and temperature in the nozzle that can affect to the drug stability and adsorption effect at the air–liquid interface upon the drying of droplets [58]. First, the physicochemical properties of INH@MIL-100 NPs dispersed in the Ma solution were evaluated to verify their colloidal stability. The INH@MIL-100 NPs in Ma solution maintained their size in the nanometric range (127 ± 27 nm) as well as their ζ-potential in negative values (−22 ± 4 mV). The INH@MIL-100:Ma ratio was chosen after some preliminary spray-drying proofs of MIL-100 NPs in Ma solution. For that, three distinct MIL-100:Ma ratios (1:5, 1:10, 1:100) were assayed and the spray-drying process was carried out as described in our previous work [19], except the inlet temperature (T_inlet_: 170 ± 2 °C) was higher than that used previously (T_inlet_: 160 ± 2 °C). The results showed that the MS had a rounded and hollow shape, and presented characteristic porosity (see Figure 4). 

As can be seen in Figure 4, the MS porosity appearance decreased as the Ma content increased. All the obtained powder samples presented a similar size in the range of 1.8–2.2 µm. Initially, the MS with the 1:5 ratio were discarded since that MS were more agglomerated compared to the other powders (Figure 4). Finally, we chose the sample with the 1:10 ratio because it had a higher NPs load and a more homogeneous appearance than MS with the 1:100 ratio.

**Figure 5:**
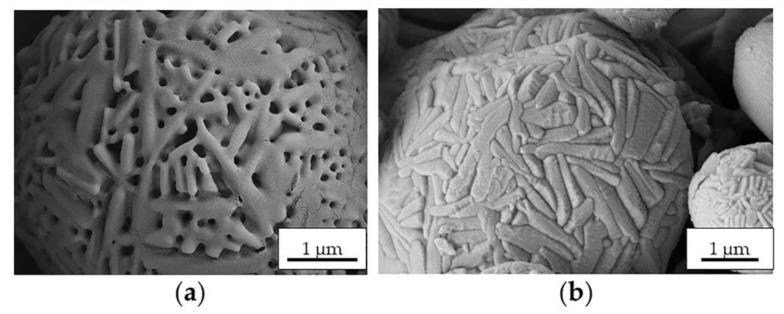
SEM microphotographs of Ma MS prepared at (**a**) 170 °C and (**b**) 160 °C.

For caution, the T_inlet_ of the INH@MIL-100 microencapsulation process was adjusted to 160 °C due to the INH presence in the vehicle (INH melting temperature: 173 °C). Control Ma MS were prepared at 160 °C as well. The surface morphologies of the Ma MS prepared at both T_inlets_ (170 and 160 °C) are different (see Figure 5): Ma MS prepared at 170 °C have a more porous structure (Figure 5a) than those obtained at 160 °C (Figure 5b). This could be explained by the different T_inlet_ used in the spray-drying process. At 170 °C, the water evaporation during the MS preparation was more abrupt and effective, generating pores in greater quantity and larger sizes, meanwhile the Ma stood more dry and compact compared with the sample obtained at 160 °C.

**Figure 6:**
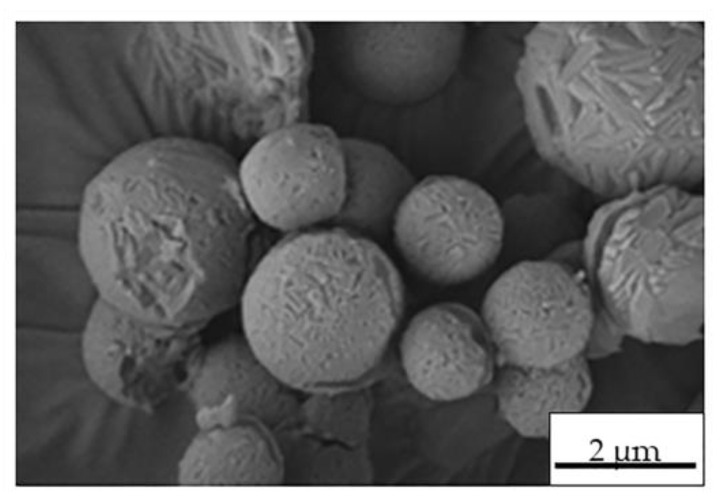
SEM microphotograph of Ma-INH@MIL-100 MS.

Therefore, the INH@MIL-100 NPs were spray-dried using Ma as the excipient and by employing the conditions that are indicated in Section 3.3. The resulting process yield was high (74.5 ± 3.5 wt%) and the Ma-INH@MIL-100 MS were non-aggregated, exhibiting a well-defined and spherical shape, as well as smooth surface (Figure 6).

**Figure 7:**
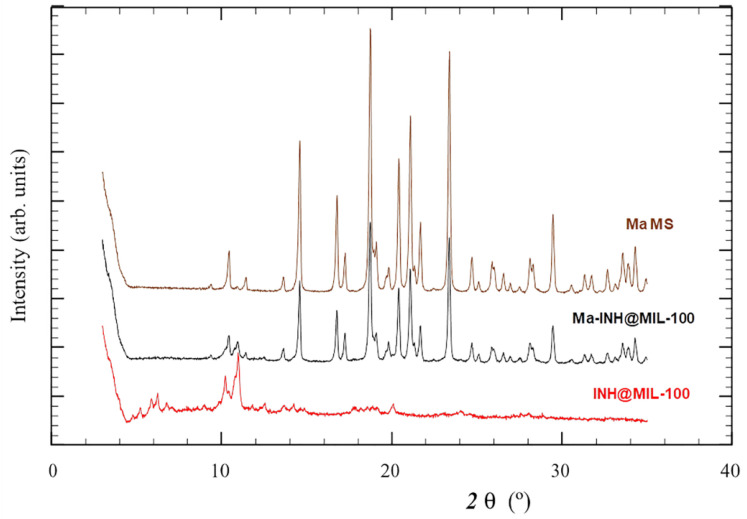
PXRD patterns of INH@MIL-100, Ma-INH@MIL-100 MS, and Ma MS (control formulation included for comparison purposes).

The crystalline structure of the MIL-100 material was kept on the MS formulation, as evidenced by the presence of the characteristic Bragg peaks of the MIL-100 structure (Figure 7). In addition, the typical diffraction peaks of Ma were present in the MS.

The compositional analyses of the different MS were carried out by means of elemental analysis (Table 2), determining the content of INH, MIL-100, and Ma by measuring the nitrogen, iron, and carbon, respectively. Additionally, it is important that their comparison with the theoretical values of these characteristic elements was employed to prepare the powder samples. Experimental values agreed with the theoretical values, confirming that the final composition of the prepared formulations maintained the amounts of the estimated elements. 

To evaluate the potential interest of Ma-INH@MIL-100 MS for pulmonary administration, their aerodynamic and physical properties were characterized (Table 3). Traditionally, diameters between 1 and 5 µm are associated with deep lung deposition; however, the geometric and aerodynamic diameters of our formulation (1.4 ± 0.4 and 0.422 ± 0.007 µm, respectively) could be considered suitable for pulmonary administration since other formulations with similar characteristics reached the deep lung in previous in vivo studies [56,59], for instance, as it was demonstrated by our research group for the Ma-MIL-100 MS in Wistar–Kyoto rats [19]. It should be noted that the low aerodynamic diameter (0.422 ± 0.007 µm, Table 3) was related with the hollow nature (ca. 70–90%) of the MS. As expected, the apparent density of Ma-INH@MIL-100 MS (0.52 ± 0.01 g·cm^−3^) was higher than that of Ma MS (0.43 ± 0.01 g·cm^−3^) considering the INH@MIL-100 cargo (Table 3). The estimated low apparent density, jointed to the small diameters for the Ma-INH@MIL-100 MS (0.52 ± 0.01 g·cm^−3^) was related to a good behavior of the aerodynamic flow, as previously demonstrated for other formulations [19,60,61]. The real density and aerodynamic diameter values of Ma-INH@MIL-100 MS were in the same range as previously obtained for Ma-MIL-100 MS [19] and other types of hollow particles that demonstrated to be acceptable for pulmonary administration [29,30].

Overall, Ma-INH@MIL-100 MS presented suitable characteristics for drug delivery to the deep lung. The selection of Ma between different excipients resulted in good results, as has been previously seen for other types of NPs, i.e., solid lipid nanoparticles (SLN) and chitosan NPs (CS NPs) [59,62].

### 2.3. Ma-INH@MIL-100 MS: Colloidal and Chemical Stability, and INH Release

**Figure 8:**
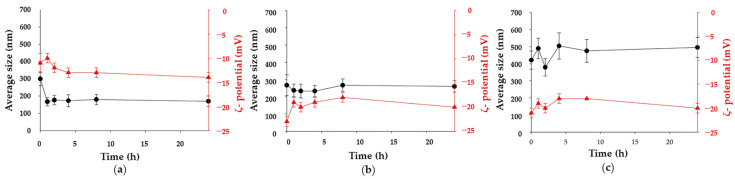
Colloidal stability of INH@MIL-100 NPs following their release from Ma MS in (**a**) water, (**b**) PBS, and (**c**) SLF, showing NPs’ size (black, left) and surface charge (red, right) vs. time (h; mean ± S.D.; *n* = 3).

**Figure 9:**
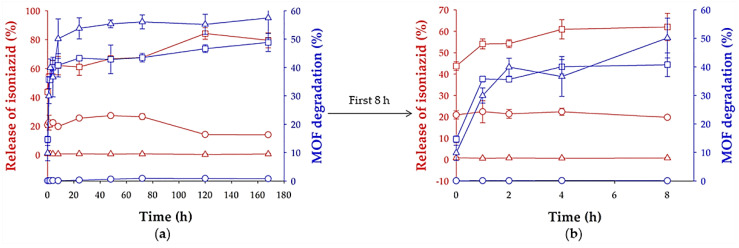
Release of INH (percentage, red) from INH@MIL-100 NPs and MOF degradation (percentage, blue) in water (circle), PBS (pH = 7.4; square), and SLF (triangle) vs. time during (**a**) 168 h and (**b**) 8 h (mean ± S.D.; *n* = 3).

Excipients can protect the nanosystems from spray-drying conditions, in particular from the heating process and intra-formulation interactions that could cause aggregation, hindering their deposition in the deep lung [56,63]. Both the release of INH@MIL-100 NPs from the MS and their colloidal stability during the release process were evaluated by monitoring the evolution of the particle size and ζ-potential of the released NPs in three simulated physiological conditions (MilliQ water; phosphate buffered saline (PBS), pH = 7.4; and simulated lung fluid (SLF) at 37 °C) for 24 h (Figure 8 and Figure 9).

**Figure 10:**
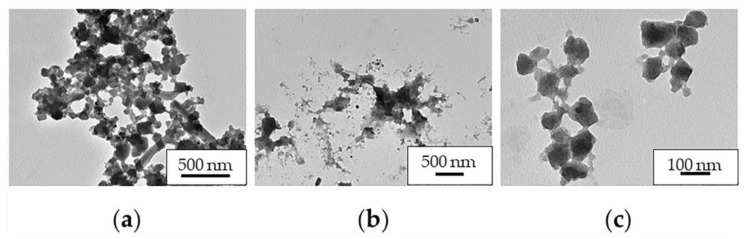
TEM microphotographs of released INH@MIL-100 NPs from Ma MS in (**a**) MilliQ water, (**b**) PBS, and (**c**) SLF.

Concerning the INH release (Figure 9), there was a rapid initial (t = 0 h) drug release of INH in MilliQ water and PBS of 21% and 44%, respectively. Then, a constant release was observed during 1 week, reaching a maximum release of 27% and 84%, at 48 h and 120 h, respectively. The incomplete release of INH could be explained by the important interactions with the MOF. Note that released INH could not be detected in SLF probably because of the formation of interactions between the drug and phospholipids of the medium, hindering its quantification by HPLC. The difference of release kinetics between MilliQ water and PBS could be related to a much faster degradation of MIL-100 NPs in PBS (1% (t = 72 h) and 49% (t = 168 h) maximum degradation, respectively; Figure 9) since phosphates from the medium can compete with carboxylates from the MIL-100 structure by the iron coordination. In SLF, the chemical stability of these NPs was similar than in PBS, although after 6 h, the MOF degradation was higher than in PBS. The difference of degradation in each medium was verified by TEM (Figure 10), where it was noticed that the INH@MIL-100 NPs maintained their initial shape in water (Figure 10a) but not in PBS with a not-defined morphology because of their higher degradation (Figure 10b). Conversely, when NPs were released in SLF, a substantial amount of particles was recovered, showing a fairly well-faceted aspect (see Figure 10c) when compared to those suspended in PBS. 

In the three tested media, the Ma excipient was quickly solubilized, releasing the INH@MIL-100 NPs from the MS. In the first hour, the NPs decreased their size in water and PBS (from 300 to 167 nm and from 267 to 237 nm, respectively), but slightly increased in the SLF (from 421 to 489 nm). In later times (up to 24 h), the MOFs were colloidally stable in water and PBS (167–180 nm and 233–267 nm, respectively), as well as in SLF where, despite their initial aggregation, the particles kept their size almost constant (over 500 nm). The ζ-potential values of released INH@MIL-100 NPs were negative and stable independently of the media: from −10 to −14 mV in MilliQ water; from −18 to −23 mV in PBS; and from −18 to −21 mV in SLF. This could be seen as an advantage because negatively charged NPs often generate lower inflammatory responses than positive NPs [64]. The increase of size (from 194.5 ± 51.9 to 460.5 ± 49.6 nm) and the reduction of the ζ-potential (from −12.2 ± 1.5 to −19.3 ± 1.2 mV) of INH@MIL-100 NPs in SLF compared to the NPs in MilliQ water could be explained by the creation of a corona constituted by proteins and lipids derived from the SLF.

These results were concordance with those demonstrated in our recent previous publication [19], in which empty MIL-100 NPs were easily released from Ma MS, maintaining their colloidal stability in the different simulated media for 24 h. In addition, the particle size evolution of INH@MIL-100 NPs was quite similar to that found for empty MIL-100 NPs, reducing their dimensions from 267 to 133 nm and from 253 to 145 nm after 1 h in MilliQ water and PBS, respectively. Similarly, in SLF, the INH@MIL-100 NPs behaved similar to their empty analogue; the MIL-100 NPs released as aggregates of around 500 nm and maintain their size for 24 h. Moreover, the surface charge of INH@MIL-100 NPs was less negative than the non-loaded MIL-100 NPs in all the media, with values of −8.0 vs. −12.2 mV (in MilliQ water); −19.5 vs. −19.8 mV (in PBS); and −18.2 vs. −19.3 mV (in SLF) for INH-loaded vs. non-loaded NPs. It is probably a consequence of the drug located both in the inner porous surface and on the outer NP surface. 

**Figure 11:**
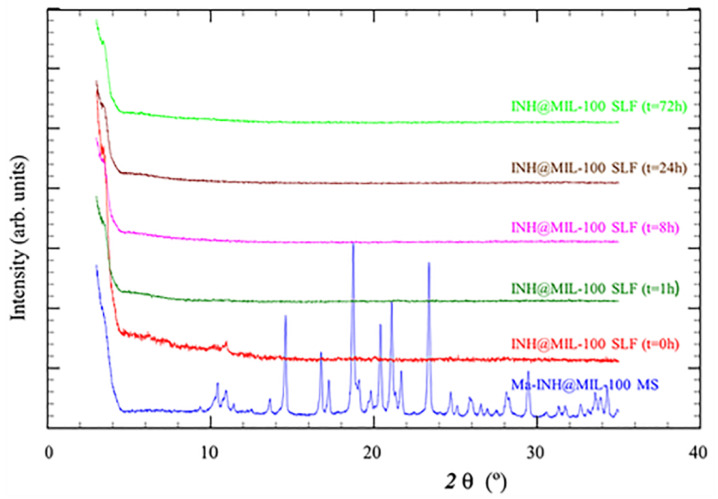
PXRD patterns of released INH@MIL-100 NPs in SLF and Ma-INH@MIL-100 MS.

The crystallinity of released INH@MIL-100 NPs was also analyzed by PXRD. In this case, the INH@MIL-100 NPs released from Ma MS in SLF (at different release times: 0, 1, 8, 24, and 72 h) were compared with the Ma-INH@MIL-100 MS (Figure 11). 

In the diffractogram, Ma diffraction peaks of the MS were observed (over 20 2 θ (°) in the blue line), showing the crystallinity of these macro-vehicles. At different release times, the Ma diffraction peaks were not appreciated because the excipient was dissolved in SLF, while the NPs were already released. As the release time progressed, the characteristic peaks of the INH@MIL-100 (over 10 2 θ (°)) were less pronounced due to the degradation of the NPs in SLF. This study confirms the crystallinity of Ma and NPs before the release, alongside the Ma solubilization and the MOFs degradation during the process, which facilitate the INH release. However, it is supposed that INH@MIL-100 NPs will be released from Ma MS in a slower manner in the alveolar fluid due to its small liquid volume (vs. experimental conditions of 4 mg·mL^−1^ of powder in release medium) [62]. Either way, it is expected that the release of INH@MIL-100 NPs from Ma MS would be good. 

### 2.4. Characterization of Test Formulations for Cell Studies

To ensure the MIL-100 and INH@MIL-100 quality for their use in cell studies, their size and ζ-potential were anteriorly determined in MilliQ water. NPs exhibited sizes in the nanometric range (101 ± 19 and 137 ± 51 nm) and negative ζ-potentials (−10 ± 11 and −18 ± 8 mV), being theoretically suitable for cell studies, as demonstrated in our previous in vivo study [19]. 

### 2.5. Cell Viability Studies

**Figure 12:**
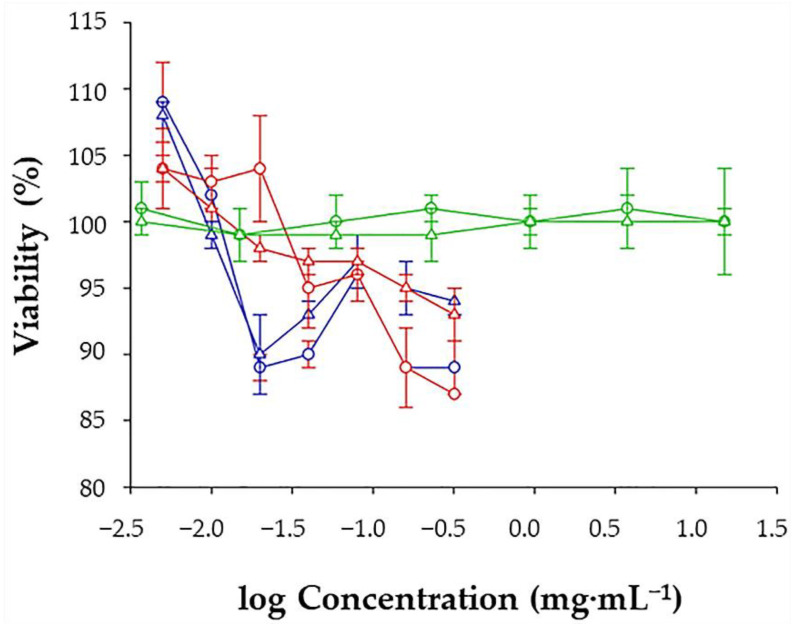
Cell viability assay 24 (circle) and 48 h (triangle) after the removal of MIL-100 (blue), INH@MIL-100 (red), and Ma (green) from A549 cells, measured by the fluorescence of CellTiter-Blue^®^ (mean ± S.D., *n* = 4).

The A549 cell line was extensively studied in vitro to evaluate the efficacy and safety of diverse drug delivery systems [65]. For this reason, it was also employed in the present viability study. Specifically, it was determined using different concentrations of MIL-100, INH@MIL-100 NPs, and Ma (see Section 3.10 for experimental details). After 24 h post-incubation, cell viability was not affected by the lower concentrations (<0.01 mg·mL^−1^) of both MIL-100 and INH@MIL-100 NPs (Figure 12). From 0.02 mg·mL^−1^, the cell viability slightly decreased with increasing NP concentration. Similar results were obtained at 48 h post-treatment. In conclusion, the cytotoxicity study shows the low toxicity of both nanosystems, indicating also that the presence of INH did not negatively affect the cell viability of the A549 cells. The results obtained here are in agreement with previous studies, in which MIL-100 NP did not induce in vitro toxicity in A549 cells (measured as cell survival/death, cell impedance, DNA damage, and ROS generation) [66]. When compared with other common nanocarriers, the viability of our systems is higher than that obtained with, for example, solid lipid NPs (SLN) of glyceryl dibehenate or glyceryl tristearate with/without rifabutin (RFB) [67], as well as with cells were treated with chitosan/tripolyphosphate (CS/TPP) NPs [65]. Finally, after 24 and 48 h post-incubation with Ma, it was confirmed that Ma was totally biocompatible with the cells (100% viability; Figure 12).

**Figure 13:**
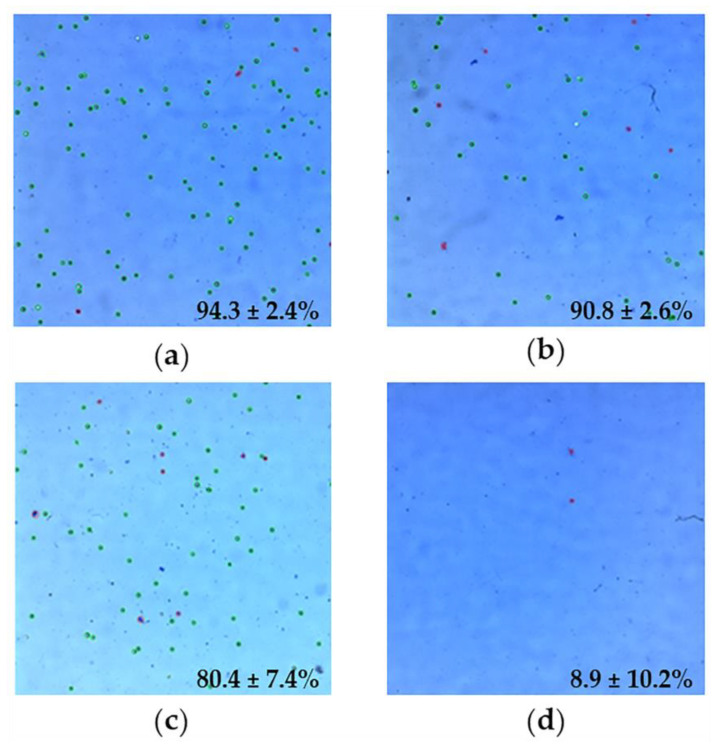
Luna II images of: (**a**) A549 cells (positive control of viability); (**b**) A549 cells treated with MIL-100 NPs; (**c**) A549 cells incubated with INH@MIL-100 NPs; and (**d**) A549 treated with Triton (negative control of viability). Living and dead cells were marked by a green or red circle, respectively. The percentages indicate the amount of living cells in each sample (mean ± S.D., *n* = 3).

A complementary cell viability assay was carried out using Luna II, where the above results were visually corroborated by the automatic counting of live cells. As shown in Figure 13, the viability of the cells incubated with MIL-100 NPs was 90.8 ± 2.6%, while the viability when employing INH@MIL-100 NPs was 80.4 ± 7.4%.

### 2.6. Intracellular Uptake and Distribution

**Figure 14:**
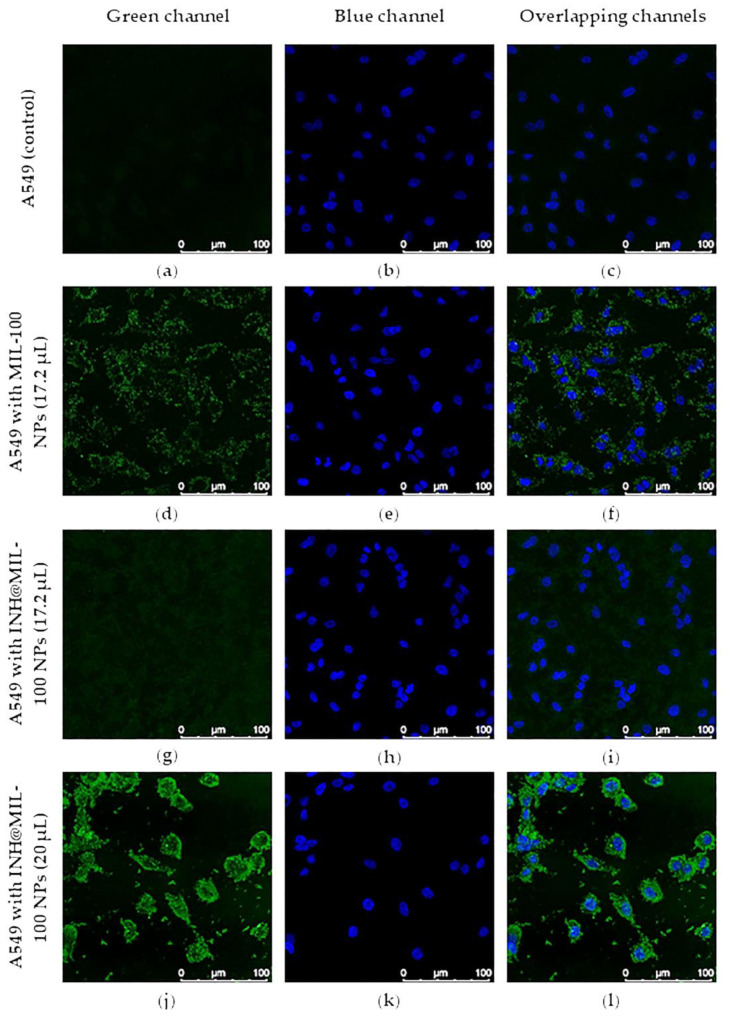
Confocal microscopy images of A549 cells: (**a**–**c**) without NPs (control); (**d**–**f**) with MIL-100 NPs (17.2 µL); (**g–i**) with INH@MIL-100 NPs (17.2 µL); and (**j**–**l**) with INH@MIL-100 NPs (20 µL; Fe self-reflection, green channel). Cell nuclei (DAPI, blue channel). Scale bar = 100 nm.

Intracellular uptake of MIL-100 and INH@MIL-100 NPs was evaluated in A549 cells by Fe self-reflection with confocal microscopy. The images of the cells (Figure 14), using 4′,6-diamino-2-phenylindole (DAPI) for nuclear staining, verified the cytoplasmic localization of MIL-100 and INH@MIL-100 NPs. It should be emphasized that the Fe self-reflection signal belonging to MIL-100 NPs (Figure 14d–f) was higher than those of INH@MIL-100 NPs (Figure 14g–i). This fact was due to a possible screening effect produced by the INH. However, the signal gain of INH@MIL-100 NPs was corrected by increasing the (i) dispersion volume of these nanosystems (20, 30, 40, and 50 µL); (ii) laser power; and (iii) number of accumulations per plane (see Figure 14j–l and Supporting Information, Figure S1).

**Figure 15:**
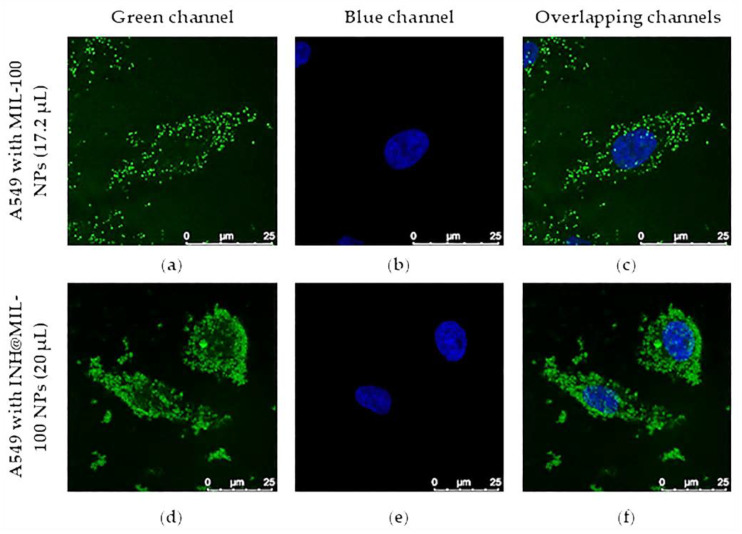
Confocal microscopy images of A549 cells (**a**–**c**) with MIL-100 NPs (17.2 µL) a d (**d**–**f**) with INH@MIL-100 NPs (20 µL; Fe self-reflection, green channel). Cell nuclei (DAPI, blue channel). Scale bar = 25 nm.

When the added dispersion volume of INH@MIL-100 NPs was progressively increased (maintaining the concentration at 3.2 mg·mL^−1^), it was detected that gradually there were more NPs outside the cells and the cells’ morphology was affected for volumes higher than 40 µL. However, these elevated volumes were only used to detect the INH@MIL-100 NPs presence inside the cells. If the results obtained from MIL-100 NPs are compared with those from INH@MIL-100 NPs using volumes of 17.2 µL and 20 µL, it could be concluded that in both cases, the nanoMOFs were perfectly internalized within the A549 cells, mainly located in the cytoplasm (Figure 15).

### 2.7. Quantification of A549 Cells Internalized with NPs: Flow Cytometry

**Figure 16:**
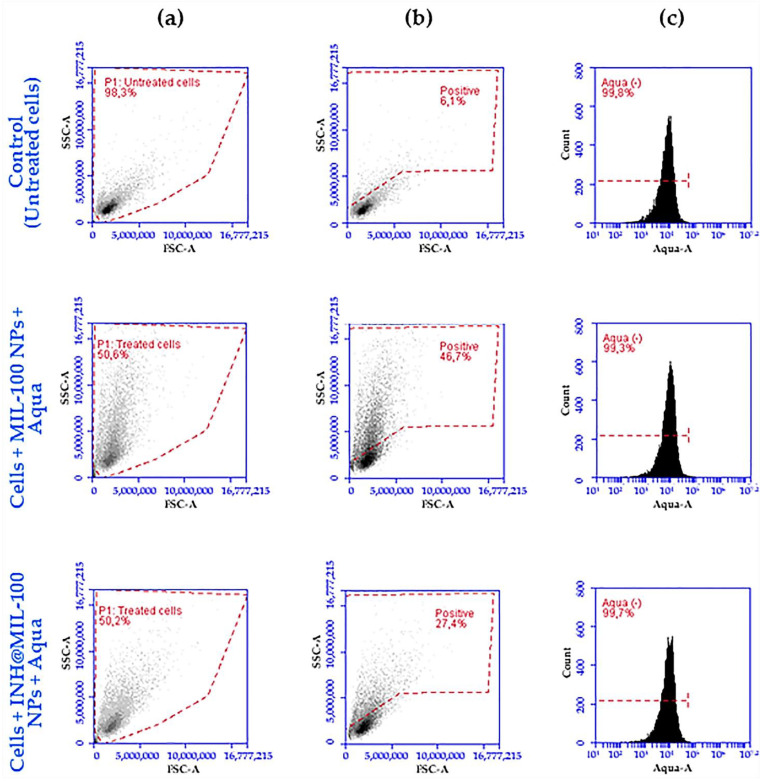
Analysis of the first replicate of MIL-100 and INH@MIL-100 NP-loaded cells by FCM: (**a**) FCM scatter plots of the total event population; (**b**) FCM scatter plots of the selected positive complexity area (the vertical axis is referred to as side scattering (SSC) and the horizontal axis is referred to as forward scattering (FSC)); and (**c**) FCM histograms of the mean fluorescence intensity of the Aqua viability reagent.

Once the internalization was visually confirmed, the quantification of that cells that had taken up NPs was carried out by flow cytometry (FCM). The study was based on a complexity analysis comparing over 10,000 events before and after the uptake of NPs. In this case, cells containing INH@MIL-100 NPs were more complex than cells containing MIL-100 NPs and these are more complex than the control (cells without NPs). In Figure 16, the first column of the FCM scatter plots represents the total population of the analyzed events. The second column of the FCM scatter plots, where the debris (apoptotic cells, membrane fragments, etc.) are excluded, corresponds with those events that present positive cellular complexity. The third column of the FCM histograms represents the mean of the fluorescence intensity (MFI) of the Aqua viability reagent to verify the viability of those selected events with positive complexity.

For the replicates of control (untreated cells) FCM scatter plots, almost 100% of the total events were analyzed (93.5 ± 4.5%) and compared to those of the cells treated with MIL-100 NPs (68.8 ± 15.9%) and INH@MIL-100 NPs (50.0 ± 3.4%; see Figure 16a and Supporting Information, Figures S2a and S3a). The decrease of this percentage was due to the exposure of A549 cells to the MIL-100 and INH@MIL-100 NPs, leading to a number of events belonging to the debris which had to be excluded for the analysis of the cellular complexity. In the FCM scatter plot, where the area of cellular complexity is determined, the control showed low values of complexity, as expected (7.4 ± 1.3%; see Figure 16b and Supporting Information, Figures S2b and S3b), verifying that the analyzed cells were found without NPs. However, in the FCM scatter plots of the treated A549 cells, they showed a higher percentage of cellular complexity compared to the control. In the case of treatment with MIL-100 NPs, positive complexity values (43.8 ± 5.0%) were obtained (see Figure 16b and Supporting Information, Figures S2b and S3b). Hence, we could assume that this cell’s percentage was positive for the presence of this nanosystem. In the case of cells treated with INH@MIL-100 NPs, positive complexity values (25.5 ± 1.8%) were also obtained but they were lower than those relative to the previous nanosystem (see Figure 16b and Supporting Information, Figures S2b and S3b). Therefore, these cells’ percentage was positive for the presence of INH@MIL-100 NPs. Although the detector was not sensitive enough to determine the difference in the complexity between control cells and NP-internalized cells due to the small size of the nanosystems, logical results were obtained. Then, it was thought that these experimental values underestimate NPs’ uptake, especially in the case of the INH-loaded nanosystem (probably as a consequence of a drug interference in the analysis method). In fact, a good uptake of both systems was confirmed anteriorly by confocal study, corroborated by Wyszogrodzka-Gaweł’s study [45], in which the authors also demonstrated that the INH-loaded Fe-MIL-101-NH_2_ were captured in greater quantities compared to the non-loaded NPs. 

Finally, it is worth highlighting the lack of cytotoxicity in the all cases, as seen in the FCM histograms (Aqua (−); see Figure 16c and Supporting Information, Figures S2c and S3c). In addition, an important viability of A549 cells was confirmed with both MIL-100 and INH@MIL-100 NPs, as can also be seen in the FCM histograms (99.4 ± 0.2% and 99.6 ± 0.2%, respectively), in comparison with the control (99.7 ± 0.1%; see Figure 16c and Supporting Information, Figures S2c and S3c), which was higher for the INH-loaded NPs than for the non-loaded nanosystems.

## 3. Materials and Methods

### 3.1. Materials

Trimesic acid (H_3_BTC, 95%, Molecular Weight-MW: 210.14 g·mol^−1^), iron(III) chloride hexahydrate (FeCl_3_·6H_2_O ≥99%, MW: 162.20 g·mol^−1^), isoniazid (INH, ≥99%, MW: 137.14 g·mol^−1^), D-mannitol (Ma, ≥98%, MW: 182.17 g·mol^−1^), α-cyclodextrin (α-CD, ≥99%, MW: 972.84 g·mol^−1^), dextran (Dex, ≥99%, MW = 72,200 g·mol^−1^), D-(+)-mannose (Man, ≥99%, MW: 180.16 g·mol^−1^), D-(+)-trehalose dihydrate (Tre, ≥99%, MW: 378.33 g·mol^−1^), D-sorbitol (Sor, ≥98%, MW: 182.17 g·mol^−1^), D-(−)-fructose (Fru, ≥99%, MW: 180.16 g·mol^−1^), L-glutamine, sodium dodecyl sulphate (SDS), and phosphate-buffered saline tablet (PBS, pH = 7.4) were acquired from Sigma Aldrich (Madrid, Spain). The A549 cell line was obtained from ATCC (Manassas, VA, USA). Dulbecco’s Modified Eagle Medium (DMEM), fetal bovine serum (FBS), trypsin-EDTA (0.05%), and Fluoromount^®^ were purchased to Gibco^TM^ (ThermoFisher Scientific, Madrid, Spain). Potassium fluoride (KF, 99%, MW: 58.09 g·mol^−1^) was obtained from Acros Organics^TM^ (Madrid, Spain). Triton^®^ X-100 (molecular biology grade) was acquired from Scharlab S.L. Laboratories (Barcelona, Spain). CellTiter-Blue (AlamarBlue^®^) was obtained from Promega (Fitchburg, MA, USA). The LIVE/DEAD™ Fixable Aqua Dead Cell Stain Kit was acquired from Invitrogen^TM^ (Waltham, MA, USA). 4′,6-diamino-2phenylindole (DAPI) was obtained from Emp-Biotech (Berlin, Germany). Trypan Blue was obtained from Logos Biosystems (Sainghin-en-Mélantois, France). Neutral buffered formalin (10% *v/v*) was purchased from Bio-Optica (Milan, Italy). MilliQ water was obtained by filtration (filters 0.2 µm, Millex^®^-GN, Millipore Iberica, Madrid, Spain). Ethanol (96%) was purchased from Labkem (Murcia, Spain). Curosurf^®^ (pig lung surfactant, 80 mg·mL^−1^ of pulmonary phospholipids) was facilitated by Professor Almeida (of the University of Lisbon, Portugal) who obtained it from Angelini Pharmacêutica, Lda. (Lisbon, Portugal).

All materials were commercially obtained and used without further purification.

### 3.2. Synthesis of MIL-100(Fe) and INH Encapsulation

MIL-100(Fe) NPs were synthesized following a previously described protocol [26]. Briefly, 6.0 mmol of FeCl_3_·6H_2_O and 4.0 mmol of H_3_BTC were dissolved in 30 mL of distilled water. The reaction was carried out by the microwave-assisted synthesis Mars-5 instrument, CEM (Midland, ON, Canada), with 130 °C over 30 s; then maintained at this temperature for 5 min and 30 s; and cooled down to room temperature (RT). The obtained NPs were centrifuged at 10,500 rpm for 25 min and then purified with both 20 mL of water (6-fold) and ethanol (1-fold) in a Thermo Scientific Heraeus Megafuge 16R Centrifuge (Loughborough, UK). After, the NPs were stored in ethanol [19]. 

For the INH encapsulation in MIL-100(Fe) NPs, a 0.15 M INH solution in water was firstly prepared. A MIL-100(Fe) NPs suspension (0.029 mmol dried powder in 500 μL of MilliQ water) was mixed with 0.6 mL of INH solution under stirring for 4 h. The obtained INH@MIL-100 solid was recovered by centrifugation (14,500 rpm for 10 min) and re-suspended in a new solution of INH, repeating the same procedure. The amount of INH incorporated in MIL-100(Fe) NPs was determined by high performance liquid chromatography (HPLC) and thermogravimetric analysis (TGA). The HPLC conditions were as follows: mobile phase was a 98:2 solution (*v/v*) of PBS (0.02 M, pH = 6.8) and methanol (MeOH). The volume of injection was set at 30 µL, the flow rate was of 1 mL·min^−1^, and the column temperature was fixed at 25 °C. The curve of the standard calibration exhibited a good correlation coefficient (≥0.99). The standard solution’s chromatogram presented a retention time of 8.77 min, identified as INH (λ_max_ at 266 nm). TGA were performed in an SDT Q-600 thermobalance (TA Instruments, New Castle, DE, USA) using a general heating profile (from 30 to 600 °C), with a heating rate of 5 °C·min^−1^, under air employing a flux of 100 mL·min^−1^. 

### 3.3. Preparation of MS

Seven saccharides (Ma, α-CD, Dex, Man, Tre, Sor, and Fru) were chosen as spray-drying excipients. They belong to the World Health Organization (WHO) Model List of Essential Medicines [68] and/or to the Inactive Ingredient Search for Approved Drug Products of the Food and Drug Administration (FDA) [69], and are employed in medical applications, healthcare, and in the food and beverage industry [70]. Solutions of these excipients were spray-dried by a simple technique using a Buchi^®^ Mini Spray Dryer B-290 (Flawil, Switzerland). The total solids content (t.s.c.) was set at 3.7 wt%; the spray-drying conditions were of the inlet temperature (T_inlet_) 102 and 160 °C; aspirator: 70%; nozzle cleaner: 5 (diameter of 0.7 mm); feed rate: 2 mL·min^−1^; air flow rate: 400 Nl·h^−1^; and the resulting outlet temperature (T_outlet_) was 61–94 °C. The obtained dried powders, consisting of MS, were collected and stored at RT in a desiccator until use. 

Once the Ma was selected as the best spray-drying excipient, 190 mg of INH@MIL-100 NPs was dispersed in 55.48 mL (34.1 mg·mL^−1^) of an Ma aqueous solution, employing an ultrasound tip (Ultrasonic Processor UP400 S—Hielscher 700 W Digital Sonifer, Teltow, Germany, at 10% amplitude and 30 s of time, in addition to two more pulses of 1 s using a water-ice bath) and an ultrasonic bath Branson 1210 (North Hampton, NH, USA) over the course of 15 min. Particle size and ζ-potential were determined by Dynamic Light Scattering (DLS) and Laser Doppler Anemometry (LDA) using a Zetasizer (Nano-ZS Nano-Series, Malvern Instruments, Malvern, UK) fixed at 25 °C. The physicochemical properties of these INH@MIL-100 NPs were examined in triplicates (*n* = 3). Afterwards, the dispersion was spray-dried by a simple technique using a Buchi^®^ Mini Spray Dryer B-290 (Flawil, Switzerland) to obtain Ma-INH@MIL-100 MS. The employed parameters to obtain the MS were of the 1:10 (*w/w*) INH@MIL-100:Ma ratio; t.s.c.: 3.7 wt%; T_inlet_: 160 ± 2 °C; aspirator: 70%; nozzle cleaner: 5 (diameter of 0.7 mm); feed rate: 2 mL·min^−1^; air flow rate: 400 NI·h^−1^ [19]; and the resulting T_outlet_ of 92–94 °C. The powders were collected and stored at RT in a desiccator until use. 

The spray-drying process yield (PY) was calculated by employing the following formula:

(1) $$PY\;\left(\%\right)=\frac{MS\;weight}{t.s.c.\;weight}\;\times \;100$$

### 3.4. Characterization of MS

The morphology of the MS was characterized by Scanning Electron Microscopy (SEM) using an ULTRA PLUS microscope (Zeiss, FESEM Ultra-Plus, Germany) at 3 KV. Samples were located over stubs using a double-sided adhesive graphite disc and coated with a layer of 10 nm of iridium using an Emitechk 550 Sputter Coater (London, UK). This process was also employed to obtain their Feret diameters (spaces between two tangents on opposite sides of a MS), which were obtained by measuring over the MS SEM images using the program z-SmartTiff (*n* = 50). Then, the geometric diameters were calculated as the averages of the obtained Feret diameters (μm). Apparent densities were calculated after submitting a powder to mechanical tapping with the device Tecnociencia (A Coruña, Spain). For that, a powder sample of known weight was introduced in a test tube of 10 mL into this apparatus, which was previously calibrated at 30 tap·min^−1^, and was submitted to simultaneous rotating and vertical movement. The volume of the powder was checked every 5 min until it became constant (*n* = 3). As the process was carried out by triplicates, the average of the three values was used to obtain the apparent densities. To obtain the theoretical aerodynamic diameter, the following formula was used:

(2) $$D_{aer}=D_{g}\sqrt{\frac{\rho_{real}}{\rho_{0}\;\lambda }}$$

where $\rho_{0}$ is 1 g·cm^−3^, $D_{g}$ is the geometric diameter (result of the average of the Feret diameters (µm)), $\rho_{real}$ is the real density of the MS (g·cm^−3^), and λ is the dynamic shape factor of the MS, with its value of 1 in spherical MS or 2 in irregular MS [71,72,73]. 

### 3.5. Study of Composition and Structural Integrity of Ma-INH@MIL-100 MS 

Once the INH@MIL-100 NPs were incorporated inside the Ma MS, the Ma-INH@MIL-100 MS were analyzed with respect to their composition and structural integrity. Powder X-ray diffraction (PXRD) patterns of samples were collected in an Empyream Panalytical diffractometer equipped with a PIXcel3D detector and copper radiation source (Cu Kα, λ = 1.5406 Å), operating at 45 kV and 40 mA. Profiles were generally collected in the 3° < 2θ < 35° range with a typical step size of 0.013° and 40 s of acquisition. N_2_ sorption isotherms were obtained at 77 K using an AutosorbQ2 (Quantachrome Instruments, Boynton Beach, FL, USA). Before to the measurement, samples were evacuated at 130 °C for 3 h. Specific surface areas were determined by applying Brunauer, Emmett, and Teller equation (BET) in the relative pressure interval of *p*/*p*_0_ = 0.01–0.3 (wherein *p*_0_ is the saturation pressure). Thermogravimetric analyses (TGA) were carried out in an SDT Q-600 thermobalance (TA Instruments, New Castle, DE, USA), with a general heating profile from 30 to 600 °C and a heating rate of 5 °C·min^−1^ under air, using a flux of 100 mL·min^−1^. Elemental analyses (EA) were determined using FLASH 2000 (Thermoscientific, Waltham, MA, USA). Inductively coupled plasma atomic emission spectroscopy (ICP-OES) analyses were performed in a Perkin Elmer Optima 7300 DV (Madrid, Spain). 

### 3.6. Ma-INH@MIL-100 MS: Colloidal and Chemical Stability, and INH Release

A quantity of 4 mg of Ma-INH@MIL-100 MS was incubated in 1 mL of different media (MilliQ water, phosphate-buffered solution (PBS, pH = 7.4), and SLF) under bidimensional stirring at 37 °C to examine the physicochemical characteristics of the released INH@MIL-100 NPs (particle size, surface charge, and colloidal stability), as well as the drug release profiles. At different times (0, 1, 2, 4, 8, and 24 h), an aliquot of 50 µL of the released INH@MIL-100 was suspended in 950 µL of its respective medium and their physicochemical properties were characterized. Particle size and ζ-potential were determined by Dynamic Light Scattering (DLS) and Laser Doppler Anemometry (LDA) using a Zetasizer (Nano-ZS Nano-Series, Malvern Instruments, Malvern, UK) fixed at 37 °C. The remaining sample was centrifuged with a Beckman Coulter^TM^ Microfuge^®^ 22R Centrifuge at 14,500 rpm for 10 min (Hyland Scientific, Stanwood, WA, USA). The recovered solid was measured by XRPD and, in order to quantify the H_3_BTC and INH, the supernatant was analyzed by HPLC using a reversed phase HPLC system Jasco LC-4000 series equipped with a PDA detector MD-4015 and a multisampler AS-4150 controlled by ChromNav software (Jasco Inc, Madrid, Spain). A Purple ODS reverse-phase column (5 µm, 4.6 × 150 mm, Análisis Vínicos, Tomelloso, Spain) was employed. For the H_3_BTC, the mobile phase used consisted of 50:50 solution (*v/v*) of PBS (0.02 M, pH = 2.5) and MeOH. For the INH, the mobile phase consisted of 98:2 solution (*v/v*) of PBS (0.02 M, pH = 6.8) and MeOH. The injection volume was set at 30 µL (with a flow rate of 1 mL·min^−1^) and the column temperature was fixed at 25 °C. The standard calibration curve showed a good correlation coefficient of ≥0.99. The chromatogram of the standard solutions showed a retention time of 3.51 min (identified as H_3_BTC, λ_max_ at 225 nm) and 8.77 min (identified as INH, λ_max_ at 266 nm). The studies were performed in triplicates (*n* = 3).

The morphological examination of the released INH@MIL-100 NPs was performed by Transmission Electron Microscopy (TEM; Jem-2010 Electron Microscope, Peabody, MA, USA) at 120 KV. For this purpose, an aliquot of 10 µL of the released sample was deposited on a copper grid (with carbon film) and the NPs were stained with 2% (*w/v*) phosphotungstic acid over the course of 2 min. 

### 3.7. A549 Cell Line 

A cell line of alveolar adenocarcinoma (human alveolar adenocarcinoma basal epithelial cells, A549) [74] was used as a model to evaluate the toxicity of MIL-100 NPs, INH@MIL-100 NPs, and the Ma excipient (viability study); the intracellular uptake and intracellular distribution of NPs (confocal fluorescence microscopy study (CLSM)); and the quantification of internalized A549 cells (cytometry study). The A549 cells were grown in DMEM supplemented with 200 mM of L-Glutamine (antibiotic) and 10% (*v/v*) FBS. Cells were incubated at 37 °C with a humidified atmosphere of 5% CO_2_ and 95% air. The A549 cells were employed between passages 12 and 32.

### 3.8. Preparation and Characterization of Test Formulations for Cell Studies

MIL-100 and INH@MIL-100 NPs were dispersed in MilliQ water (3.2 mg·mL^−1^) by vortex and an ultrasound tip (Ultrasonic Processor UP400 S–Hielscher 700 W Digital Sonifer, Germany) at 10% amplitude and 30 s of time, in addition to two more pulses of 1 s using a water-ice bath. MIL-100 NPs were previously washed twice with MilliQ water to remove the ethanol, employing a Beckman Coulter^TM^ Microfuge^®^ 22R Centrifuge (Hyland Scientific, Stanwood, WA, USA).

The physicochemical properties of MIL-100 and INH@MIL-100 NPs were characterized. Particle size and ζ-potential were determined by Dynamic Light Scattering (DLS) and Laser Doppler Anemometry (LDA) using a Zetasizer (Nano-ZS Nano-Series, Malvern Instruments, Malvern, UK) fixed at 25 °C. The physicochemical properties were analyzed in triplicates (*n* = 3).

### 3.9. Preparation of Test-Ma Solutions for the Viability Study

A Ma solution at 15% (*w/v*) was directly prepared by dissolving the Ma excipient in supplemented DMEM under sterile conditions. Then, a serial dilution to the fourth part was made using supplemented DMEM to test the cell viability at different concentrations of the Ma solution.

### 3.10. Cell Viability Studies

The viability studies of A549 cells treated with MIL-100 and INH@MIL-100 NPs aqueous dispersions, as well as the Ma solution, were evaluated using CellTiter-Blue^®^ as a viability reagent. Two plates of 96 wells were seeded with 100 µL of A549 cells and DMEM (9000 cells/well), and they were incubated at 37 °C for 48 h, employing a humidified atmosphere of 5% CO_2_ and 95% air, to facilitate the growing and development of the cells until they were confluents. The media were replaced with 100 µL of a mixture of: (i) 1:10 (*v/v*) MilliQ water:DMEM (positive control); (ii) 1:10 (*v/v*) MIL-100:DMEM; (iii) 1:10 (*v/v*) INH@MIL-100:DMEM; (iv) 1:10 (*v/v*) Triton (1% (*v/v*)):DMEM (negative control); and (v) Ma:DMEM. Different concentrations of nanoMOFs (MIL-100 NPs and INH@MIL-100 NPs) and Ma in DMEM were tested (see Table 4).

After 4 h of incubation at 37 °C, the controls and samples were removed and the cells were washed with PBS (pH = 7.4). Then, 100 µL of fresh DMEM was added to each well. Cells were incubated for 24 and 48 h. Next, 20 µL of CellTiter-Blue^®^ was added to every well in darkness to label cell nuclei and the metabolic capacity of live cells was measured according to the manufacturer′s instructions after lysis with 3% (*w/v*) SDS. Briefly, lysates were placed into a black 96-well plate and fluorescence was measured in a microplate reader (SYNERGY H1M BioTek^®^) at 539 nm of excitation wavelength and 620 nm of emission wavelength by Gen5 Software (Image Sofware BioTek^®^).

Cell viability (percentage) compared with the control cells was calculated as follows:

(3) $$Cell\;viability\;\left(\%\right)=\frac{Sample\;fluorescence}{Positive\;control\;fluorescence}\;\times \;100$$

The study was carried out in quadruplicates (*n* = 4).

Moreover, a complementary assay to test the cell viability was performed using the Luna II instrument (Luna II^TM^ Automated Cell Counter, Logos Biosystems, Annandale, VA, USA). For this purpose, plates of 24 wells were seeded with 400 µL of A549 cells and DMEM (60,000 cells/well), and then they were incubated at 37 °C for 48 h, employing a humidified atmosphere of 5% CO_2_ and 95% air, to facilitate the growth and development of the cells until their confluence. The media were replaced with 400 µL of a mixture of: (i) 1:10 (*v/v*) MilliQ water:DMEM (positive control); (ii) 1:23 (*v/v*) MIL-100:DMEM; (iii) 1:20 (*v/v*) INH@MIL-100:DMEM; and (iv) 1:10 (*v/v*) Triton:DMEM (negative control). Originally, both nanoMOF dispersions in MilliQ water presented a concentration of 3.2 mg·mL^−1^ and posteriorly with DMEM presented a concentration of 0.14 and 0.16 mg·mL^−1^/well for MIL-100 and INH@MIL-100 NPs, respectively. Plates were incubated at 37 °C for 4 h and then cells were washed 3 times with PBS (pH = 7.4, 5 min at RT in rocking-stirring, with a 15° inclination, using VWR Rocking platform shaker 230V, Lutterworth, UK). Cells were detached by 120 µL of trypsin (5 min, 37 °C). To deactivate the trypsin, 280 µL of DMEM was added. Cells were centrifuged at 1477 rpm (Eppendorf 5415R Refrigerated Centrifuge, North Hampton, NH, USA) for 5 min, obtaining a pellet that was resuspended in 500 µL of PBS (pH = 7.4), supplemented with 10% (*v/v*) FBS. In total, 10 µL of each sample was mixed by vortex with 10 µL of 0.4% (*w/v*) Trypan Blue stain and was observed in a cell count camera to obtain the viability values by automatic image counting. The study was carried out in triplicates (*n* = 3).

### 3.11. Intracellular Uptake and Distribution

Plates of 24 wells were seeded with 400 µL of A549 cells in DMEM (60,000 cells/well) using polylysine-treated coverslips. As it was previously indicated, the cells were incubated at 37 °C for 48 h, employing a humidified atmosphere of 5% CO_2_ and 95% air, to facilitate the growth and development of the cells until their confluence. The media were replaced with 400 µL of (i) DMEM (control) and with a mixture of (ii) 1:23 (*v/v*) MIL-100:DMEM and (iii) 1:20 (*v/v*) INH@MIL-100:DMEM. Originally, both nanoMOF dispersions in MilliQ water presented a concentration of 3.2 mg·mL^−1^ and posteriorly with DMEM presented a concentration of 0.14 mg·mL^−1^/well. In the case of the drug-loaded NPs, to verify their better visualization in confocal microscopy, different volumes of 3.2 mg·mL^−1^ INH@MIL-100 NPs aqueous dispersion (20, 30, 40, and 50 µL) were employed. Cells were incubated at 37 °C for 4 h and then washed 3 times with PBS (pH = 7.4, 5 min at RT in rocking-stirring with a 15° inclination). Cells were fixed by adding 350 µL of 10% (*v/v*) neutral-buffered formalin (15 min at RT in rocking-stirring with a 15° inclination). Then, another 3 washes were carried out as previously described and the nuclei of the cells were labelled with 200 µL of a dilution 1:1000 (*v/v*) of DAPI in PBS (1 mg·mL^−1^). The excess of DAPI was removed by washing 3 times as previously mentioned. The glass coverslips were placed on glass slides employing Fluoromount^®^ aqueous mounting medium for their visualization by CLSM (confocal microscope Leica TCS SP5 X, Wetzlar, Germany), employing an objective HCX PL APO CS 63.0 x 1.30 GLYC 21 °C UV (at 63X) and a white laser. To visualize the samples, a drop of immersion oil was added (oil immersion lens HCX PL Fluotar). 

The visualization of the MIL-100 and INH@MIL-100 NPs was obtained by Fe self-reflection (λ_Ex/Em:_ 488/485–490 nm). The green color was manually set for MIL-100 and INH@MIL-100 NPs by employing the LAS AF (Leica Application Suite Advanced Fluorescence) software. To visualize the nuclei of the cells labelled with DAPI, the software was employed on other channels (λ_Ex/Em:_ 405/414–440 nm) and the color blue was fixed. Therefore, both two signals were collected using separate channels. The conditions tested by confocal microscopy are summarized in Table 5.

### 3.12. Quantification of A549 Cells Internalized with NPs: Flow Cytometry

To quantify the A549 cells internalized with nanoMOFs, a study based on flow cytometry (FCM) was carried out. Plates of 24 wells were seeded with 400 µL of A549 cells and DMEM (60,000 cells/well). As it was previously indicated, the cells were incubated at 37 °C for 48 h, employing a humidified atmosphere of 5% CO_2_ and 95% air, to facilitate the growing and development of the cells until they were confluents. The media were replaced with 400 µL of (i) DMEM (control) and a mixture of (ii) 1:23 (*v/v*) MIL-100:DMEM and (iii) 1:20 (*v/v*) INH@MIL-100:DMEM. Originally, both nanoMOF dispersions in MilliQ water presented a concentration of 3.2 mg·mL^−1^ and posteriorly with DMEM presented a concentration of 0.14 and 0.16 mg·mL^−1^/well for MIL-100 and INH@MIL-100 NPs, respectively. Cells were incubated at 37 °C for 4 h and then washed 3 times with PBS (pH = 7.4, 5 min at RT in rocking-stirring with a 15° inclination). Then, except for the control, cells were treated with 200 µL of the LIVE/DEAD™ Fixable Aqua Dead Cell Stain Kit diluted in PBS (1 µL of Aqua in 2 mL of PBS (*v/v*); 15 min at RT in rocking-stirring with a 15° inclination). The A549 cells were washed with PBS as mentioned previously and then were detached by 120 µL of trypsin (5 min, 37 °C). To deactivate the trypsin, 280 µL of DMEM was added. Cells were centrifuged at 1477 rpm (Eppendorf 5415R Refrigerated Centrifuge, North Hampton, NH, USA) for 5 min, obtaining a pellet that was resuspended in 500 µL of PBS (pH = 7.4), supplemented with 10% (*v/v*) FBS. Finally, a total of approximately 10,000 events were automatically counted by employing a cytometer model Accuri Becton Dickinson (BD Accuri^TM^, Ann Arbor, MI, USA) and were analyzed by BD sample software (BD Biosciences, San Jose, CA, USA). The quantification of cells internalized with MIL-100 and INH@MIL-100 NPs was performed by complexity, with the detector side scatter employed (SSC-A). In addition, to verify the viability of the internalized cells, the filter BP 515/20 (Waltham, MA, USA) for the Aqua viability reagent was used. The study was carried out in triplicates (*n* = 3).

## 4. Conclusions

In this work, the first line anti-TB drug INH was efficiently loaded in highly porous and biocompatible MIL-100 NPs, and was further effectively microencapsulated in Ma MS by a simple spray-drying technique, with the aim of producing a suitable pulmonary formulation and to offer a better vehicle to improve the traditional anti-TB treatment. The MS obtained in the form of dry powders present suitable characteristics for deep lung delivery, such as regarding their morphology and aerodynamic properties. Additionally, Ma-INH@MIL-100 MS are able to carry high-drug loadings, as well as can release the NPs and INH in different aqueous media. MIL-100 NPs, INH@MIL-100 NPs, and Ma demonstrated, by in vitro studies, low toxicity for the human alveolar adenocarcinoma basal epithelial cells (A549) and were efficiently internalized by them, with the main location in the cytoplasmic zone. These systems, due to their biosafety and adapted pulmonary formulation, are promising candidates for the local pulmonary treatment of infectious diseases and thus they interesting for TB.

## Figures and Tables

**Table 1:** Process yield (wt, percentage) as well as morphology and apparent density (g·cm^−3^) of control MS (dry powders) prepared with different excipients (mean ± S.D.; *n* = 3).

Control MS	Process Yield (wt, %)	Morphology	Apparent Density (g·cm^−3^)
Ma MS	58.5 ± 3.5	Spherical	0.43 ± 0.01
*α*-CD MS	74.0 ± 3.0	Less spherical	0.36 ± 0.01
Dex MS	10.5 ± 1.5	Less spherical	- ^1^
Tre MS	27.0 ± 1.0	Agglomerate	- ^1^

^1^ Not enough samples to perform this calculation.

**Table 2:** Elemental composition (weight percentage) of Ma MS, Ma-MIL-100 MS, and Ma-INH@MIL-100 MS.

Samples	Theoretical Values				Experimental Values			
	%C	%H	%N	%Fe	%C	%H	%N	%Fe
Ma MS	39.50	7.68	-	-	39.72 ± 0.24	7.50 ± 0.01	-	-
Ma-MIL-100 MS	38.89	7.08	-	2.50	39.79 ± 0.06	6.75 ± 0.07	-	2.68 ± 0.13
Ma-INH@MIL-100 MS	39.40	7.32	0.55	1.60	39.98 ± 0.09	7.24 ± 0.06	0.56 ± 0.07	1.25 ± 0.06

**Table 3:** Aerodynamic and physical properties of Ma MS, Ma-MIL-100 MS, and Ma-INH@MIL-100 MS (mean ± S.D., *n* = 3).

Sample	Geometric Diameter (µm)	Real Density (g·cm^−3^)	Apparent Density (g·cm^−3^)	Aerodynamic Diameter (µm)
Ma MS	2.3 ± 1.0	0.0666 ± 0.0001	0.43 ± 0.01	0.594 ± 0.010
Ma-MIL-100 MS	1.8 ± 0.7	0.0550 ± 0.0001	0.44 ± 0.02	0.422 ± 0.007
Ma-INH@MIL-100 MS	1.4 ± 0.4	0.0907 ± 0.0003	0.52 ± 0.01	0.422 ± 0.007

**Table 4:** Tested concentrations of nanoMOF suspensions and Ma solutions in DMEM tested in A549 cells.

NanoMOFs (mg·mL^−1^)	Ma (mg·mL^−1^)
0.32	15.00
0.16	3.75
0.08	0.94
0.04	0.23
0.02	0.059
0.01	0.015
0.005	0.0037

**Table 5:** Conditions employed in the study of the intracellular uptake and distribution of MIL-100 NPs and INH@MIL-100 NPs (3.2 mg·mL^−1^ in MilliQ water) in A549 cells.

Sample	Volume of NP Dispersions (µL)	Number of Accumulations Per Plane
Without NPs	-	2
MIL-100	17.2	2
INH@MIL-100	17.2	2
INH@MIL-100	20.0	16
INH@MIL-100	30.0	16
INH@MIL-100	40.0	16
INH@MIL-100	50.0	16

## Data Availability

Data did not present in this study are available in supplementary material.

## Supplementary Materials

The following materials are available online. Figure S1: Confocal microscopy images of A549 cells: (**a**–**c**) with INH@MIL-100 NPs (30 µL); (**d**–**f**) with INH@MIL-100 NPs (40 µL); (**g**–**i**) with INH@MIL-100 NPs (50 µL) (Fe self-reflection, green channel). Cell nuclei (DAPI, blue channel). Scale bar = 100 nm. Figure S2: Analysis of the second replicate of MIL-100 and INH@MIL-100 NPs-loaded cells by FCM: (**A**) FCM scatter plots of total event population; (**B**) FCM scatter plots of the selected positive complexity area (the vertical axis is referred to as side scattering (SSC) and the horizontal axis is referred to as forward scattering (FSC)); (**C**) FCM histograms of the mean fluorescence intensity of the Aqua viability reagent. Figure S3: Analysis of the third replicate of MIL-100 and INH@MIL-100 NPs-loaded cells by FCM: (**A**) FCM scatter plots of total event population; (**B**) FCM scatter plots of the selected positive complexity area (the vertical axis is referred to as side scattering (SSC) and the horizontal axis is referred to as forward scattering (FSC)); (**C**) FCM histograms of the mean fluorescence intensity of the Aqua viability reagent.

## Sample Availability

Not available.
