# Supplementary material for: Microencapsulated Isoniazid-Loaded Metal–Organic Frameworks for Pulmonary Administration of Antituberculosis Drugs
Source: Molecules. 2021 Oct 23;26(21):6408. doi: 10.3390/molecules26216408 (PMC8587908; doi:10.3390/molecules26216408)
Supplement: Supplementary file 1 [file molecules-26-06408-s001.zip › molecules-1395584-supplementary.pdf]

Supporting Information

Microencapsulated isoniazid-loaded MIL-100 nanoparticles (INH@MIL-100 NPs)

Cristina Fernández-Paz [1], Estefanía Fernández-Paz [1], Pablo Salcedo-Abraira [2], Sara Rojas [2], Sheila Barrios-Esteban [3], Noemi Csaba [3], Patricia Horcajada [2] and Carmen Remuñán-López [1,*]

[1] Nanobiofar Group, Department of Pharmacology, Pharmacy & Pharmaceutical Technology. Faculty of Pharmacy, University of Santiago de Compostela, Campus Vida, 15782, Santiago de Compostela, Galicia, Spain.
[2] Advanced Porous Materials Unit (APMU), IMDEA Energy Institute. Av. Ramón de la Sagra, 3, 28035, Móstoles, Madrid, Spain.
[3] Nanobiofar Group-Natural Polymers and Biomimetics (NPNB) Group. Center of Research in Molecular Medicine and Chronic Diseases (CiMUS), University of Santiago de Compostela, Campus Vida, 15706, Santiago de Compostela, Galicia, Spain.
* Correspondence: mdelcarmen.remunan@usc.es; Tel.: (+34 881815045)

Table of Contents

Figure S1. 2

Figure S2. 3

Figure S3. 4

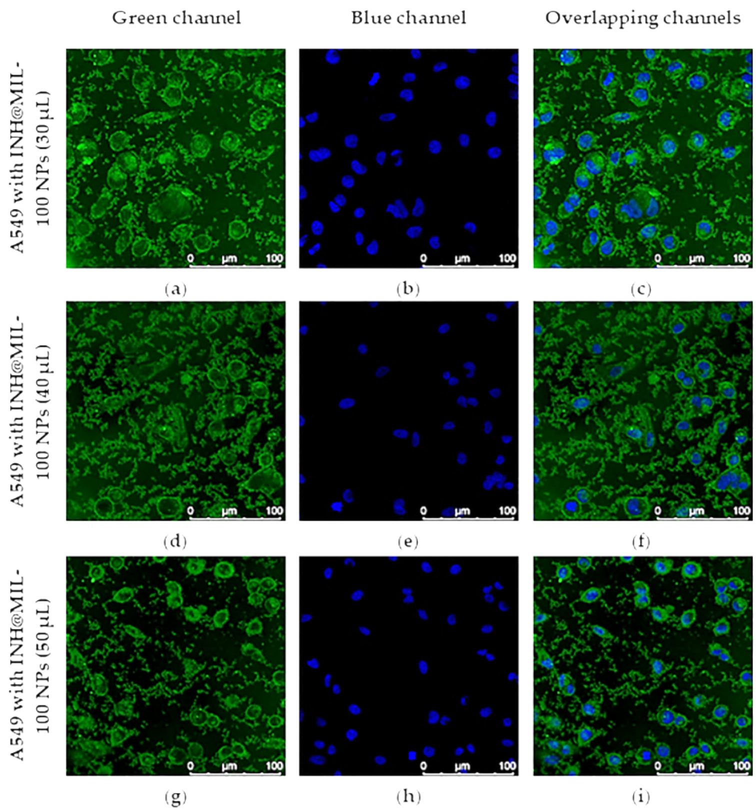

Figure S1. Confocal microscopy images of A549 cells: (**a-c**) with INH@MIL-100 NPs (30 μL); (**d-f**) with INH@MIL-100 NPs (40 μL); (**g-i**) with INH@MIL-100 NPs (50 μL) (Fe self-reflection, green channel). Cell nuclei (DAPI, blue channel). Scale bar = 100 nm.

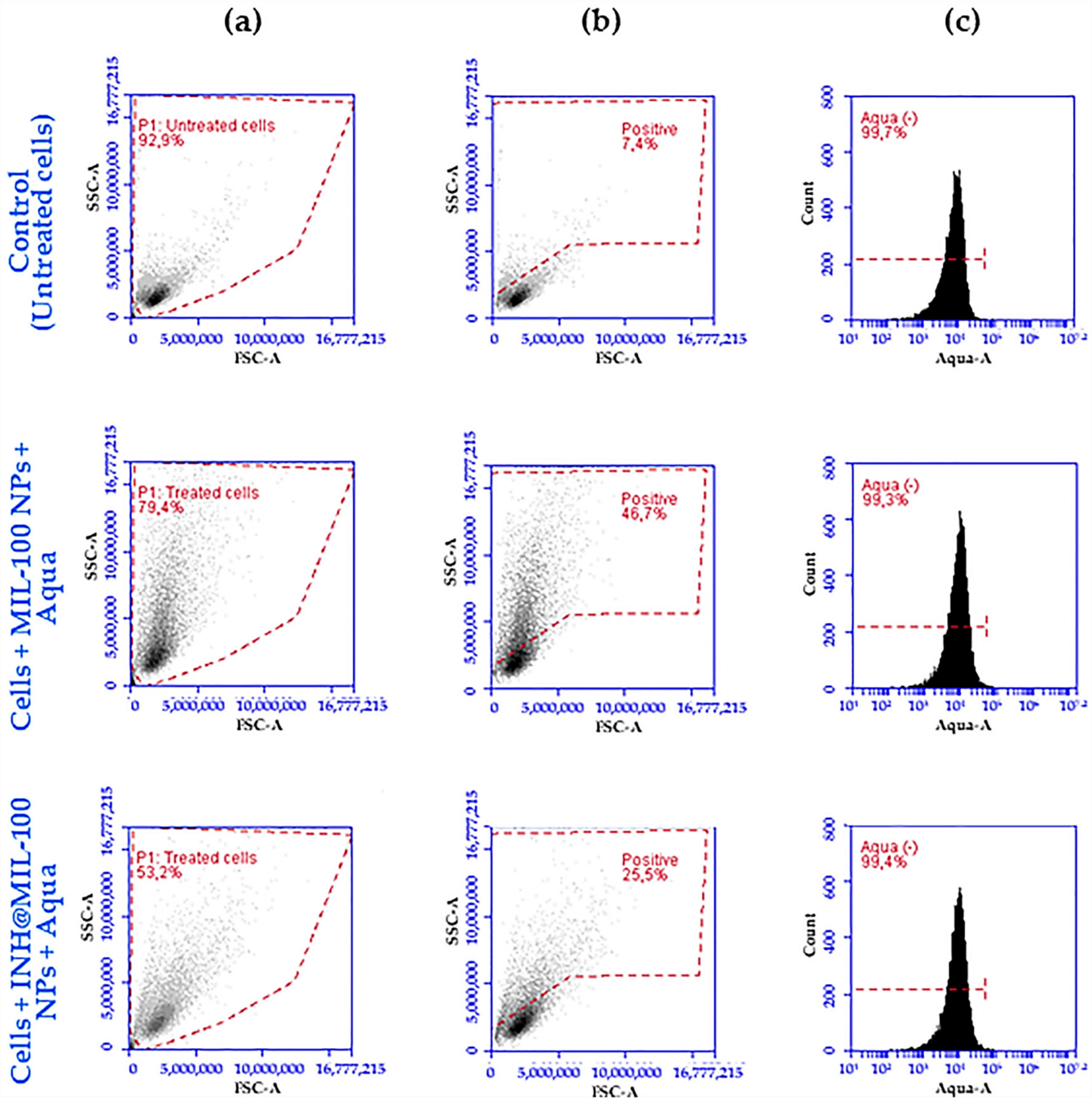

Figure S2. Analysis of the second replicate of MIL-100 and INH@MIL-100 NPs-loaded cells by FCM: (**A**) FCM scatter plots of total event population; (**B**) FCM scatter plots of the selected positive complexity area (the vertical axis is referred to as side scattering (SSC) and the horizontal axis is referred to as forward scattering (FSC)); (**C**) FCM histograms of the mean fluorescence intensity of the Aqua viability reagent.

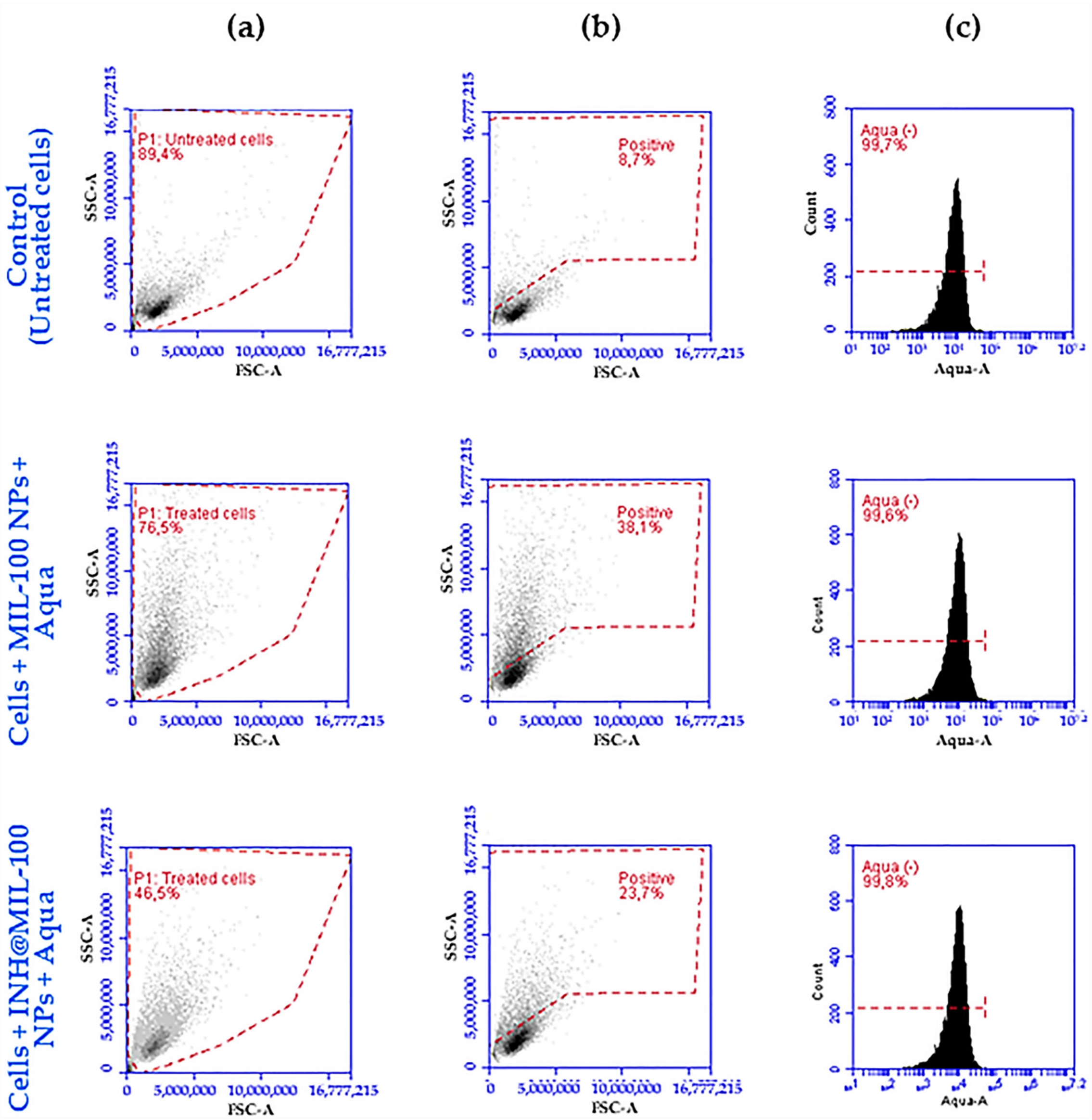

Figure S3. Analysis of the third replicate of MIL-100 and INH@MIL-100 NPs-loaded cells by FCM: (**A**) FCM scatter plots of total event population; (**B**) FCM scatter plots of the selected positive complexity area (the vertical axis is referred to as side scattering (SSC) and the horizontal axis is referred to as forward scattering (FSC)); (**C**) FCM histograms of the mean fluorescence intensity of the Aqua viability reagent.
